# Supplementary figures and images for: The effects of combining focus of attention and autonomy support on shot accuracy in the penalty kick
Source: PLoS One. 2019 Sep 23;14(9):e0213487. doi: 10.1371/journal.pone.0213487 (PMC6756529; doi:10.1371/journal.pone.0213487)

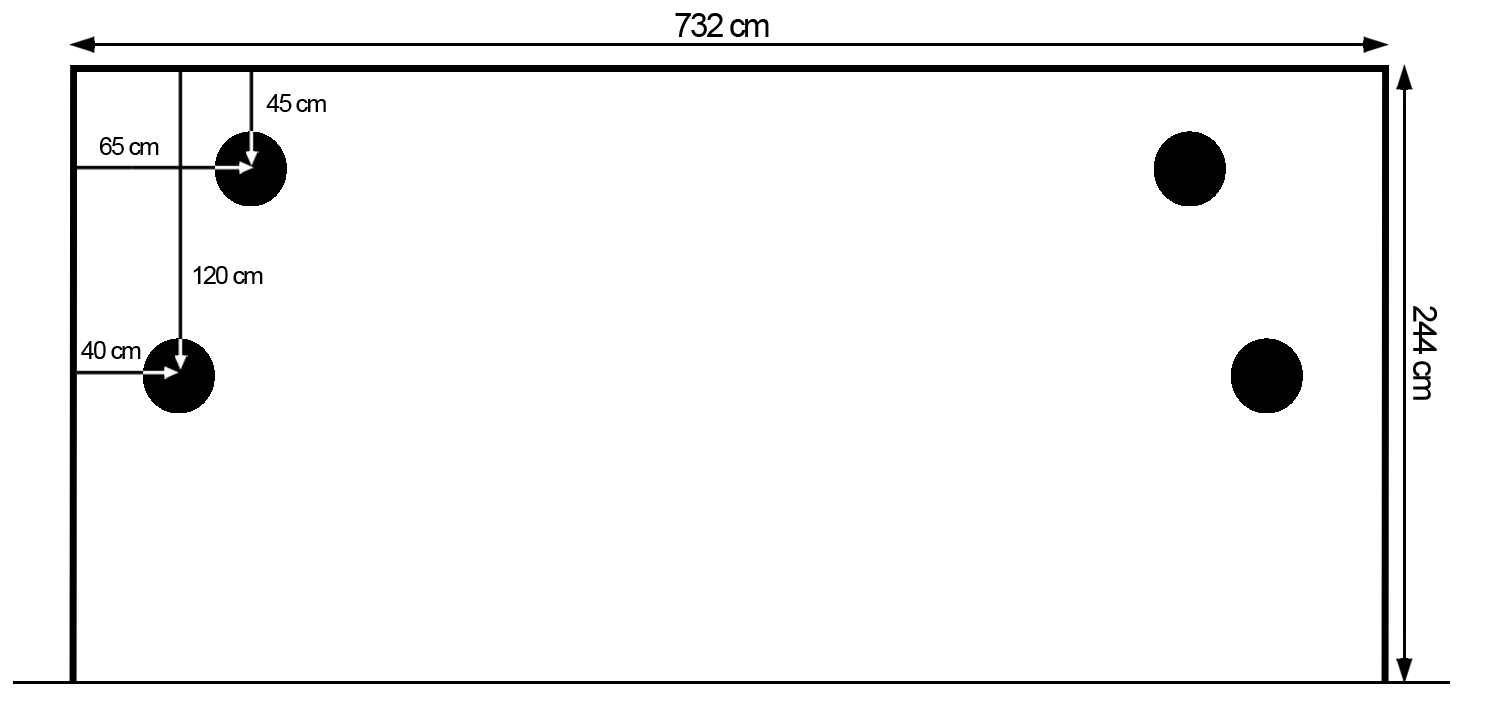

Supplement: S1 Fig — (TIF) [file pone.0213487.s001.tif]

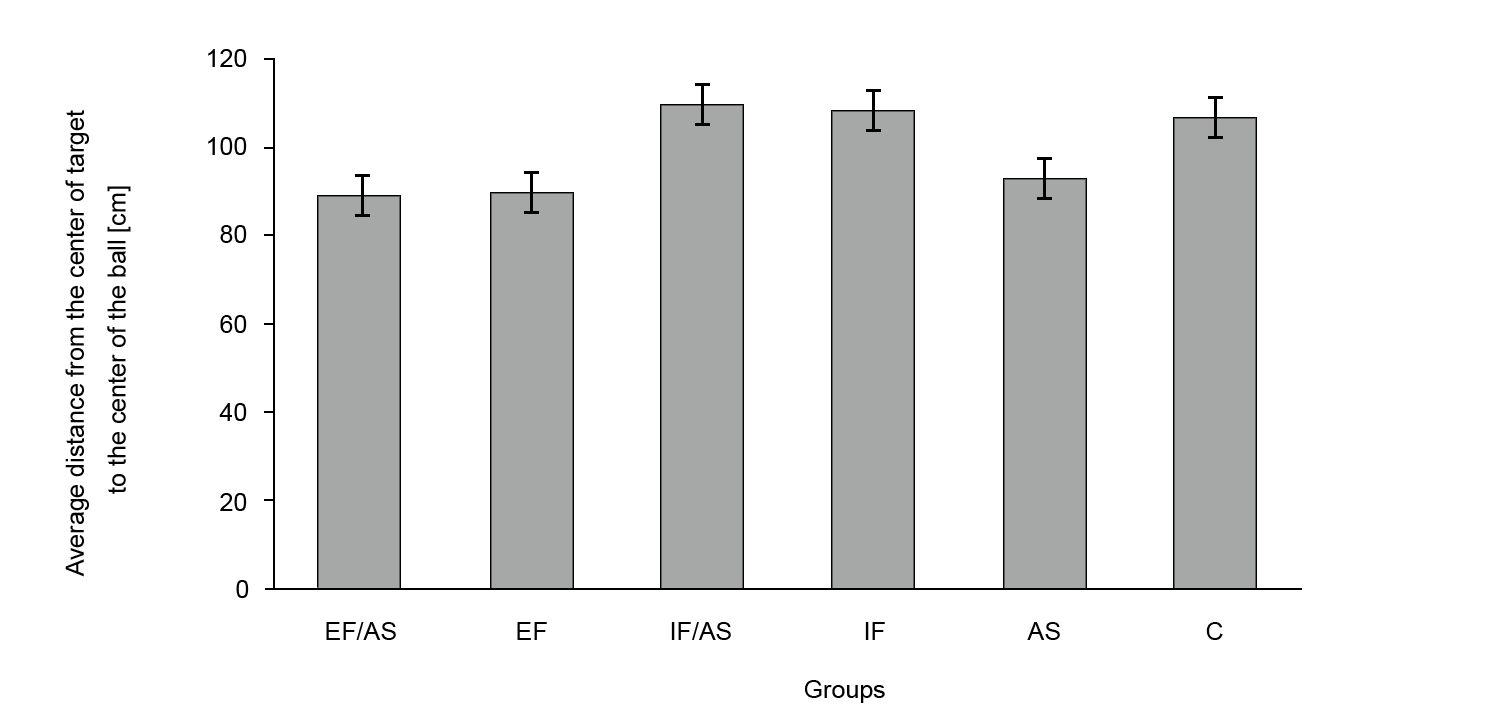

Supplement: S2 Fig — (TIF) [file pone.0213487.s002.tif]

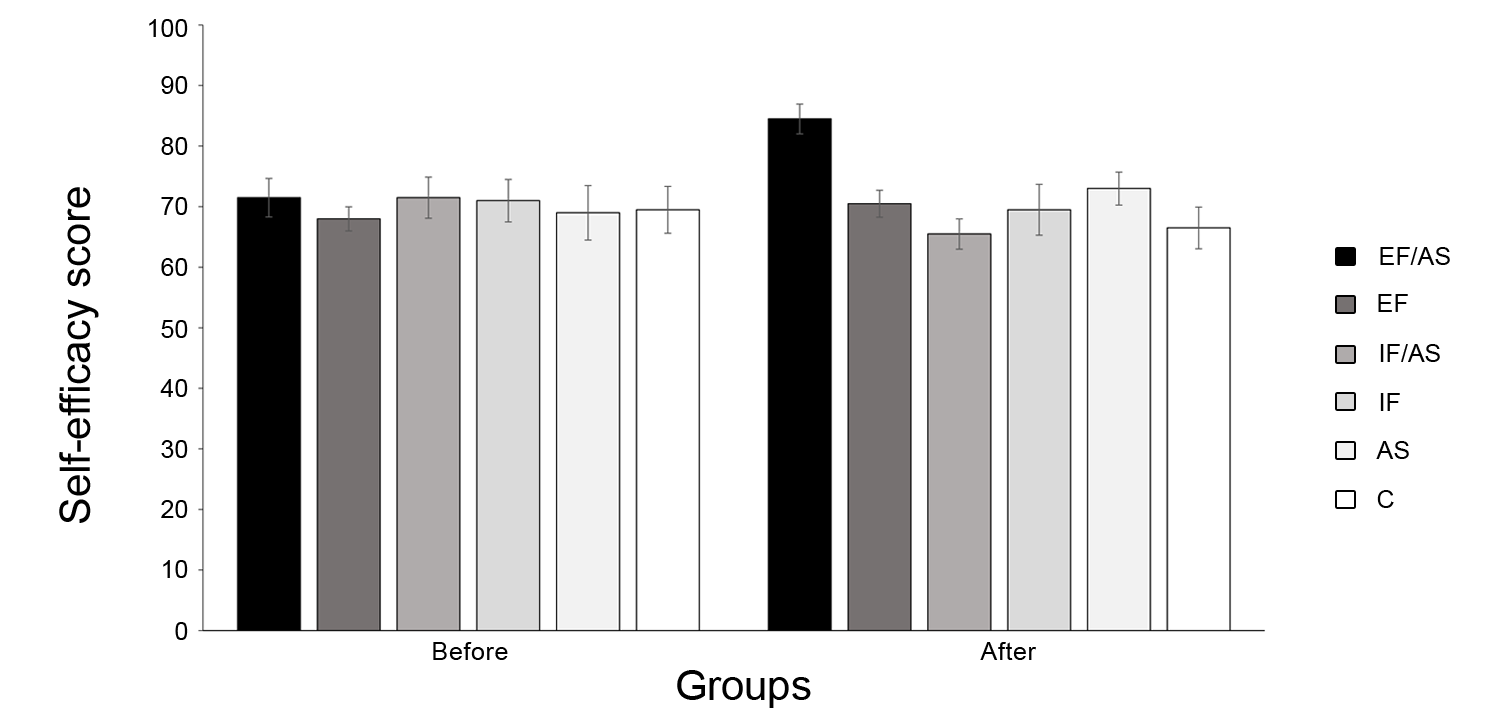

Supplement: S3 Fig — (TIF) [file pone.0213487.s003.tif]
